# Smoke and mirrors – does smoking cause discolouration of composite restorations?

**DOI:** 10.1038/s41432-023-00955-8

**Published:** 2023-11-22

**Authors:** Ranj Abdulla, Kirsty Cowan

**Affiliations:** 1 Dental Core Trainee Glasgow Dental Hospital and School, Glasgow, UK; 2 Specialty Registrar Dundee Dental Hospital and Research School, Dundee, UK

**Keywords:** Bonded restorations, Composite resin

## Abstract

**Data sources:**

Searches were carried out across PubMed, Embase, Scopus and Web of Science for studies published until 28th March 2022.

**Study selection:**

In vitro studies assessing colour stability of resin-based composites (RBCs) exposed to conventional cigarette smoke (CS), tobacco heating systems (THS) or electronic nicotine delivery systems (ENDS) were considered. Included studies used a spectrophotometer or colorimeter for assessment of discolouration and measured discolouration using CIELAB or CIEDE2000 colour difference formulas. Literature not published in English was excluded.

**Data extraction and synthesis:**

Data was extracted from thirteen studies which met the inclusion criteria. A data collection form was used to collate the extracted information on sample shape, sample diameter, sample thickness, time elapsed before smoke exposure, smoke exposure protocol, colour measurement device, sample finishing method, brushing simulation and whether exposure was to CS, THS or ENDS.

**Results:**

All 13 included studies analysed CS, four studies analysed ENDS and two studies analysed THS. A high level of variability was identified between the studies in relation to smoke exposure protocol. CS caused the highest level of discolouration of RBCs.

**Conclusions:**

This systematic review of in vitro studies found CS caused irreversible RBC discolouration. ENDS and THS caused less colour change, which could be reversed with repolishing or bleaching procedures, although evidence was limited. Further research is required to consider the long-term effect of CS, ENDS and THS on discolouration of RBCs.

## A Commentary on

**Paolone G, Pavan F, Mandurino M** ***et al***.

Colour stability of resin-based composites exposed to smoke. A systematic review. *J Esthet Restor Dent* 2023; **35:** 309–321.

**GRADE Rating**: 

## Commentary

Paolone et al. evaluated the colour stability of resin-based composites (RBCs) exposed to conventional cigarette smoke (CS), tobacco heating systems (THS) or electronic nicotine delivery systems (ENDS) through systematic review of in vitro studies^1^. In comparison to conventional cigarettes, THS heat tobacco at a lower temperature and emit a mix of nicotine and other components to be inhaled^2^. ENDS heat a liquid containing nicotine but not tobacco, and are commonly known as “vapes” or “e-cigarettes”^3^. Within the United Kingdom, there has been a decrease in conventional cigarette smoking but an increase in e-cigarette use^4^. Composite resin is a widely used dental material selected by clinicians for both aesthetic and mechanical properties^5^. Given the prevalence of smoking and the popularity of composite resin restorations, this systematic review is a welcome addition to existing literature.

Although four databases were searched, the authors only included studies published in English and did not search grey literature for relevant studies. After screening 273 studies, 13 studies were selected for data extraction of which all analysed CS, four analysed ENDS and two analysed THS. Whilst this systematic review only included in vitro studies, the majority of studies in the wider literature relating to smoking and staining of dental materials appear to be in vitro^6^.

There was high variability amongst the methodology of the included studies. Eleven studies used RBC discs and the remaining two used cavitated teeth restored with composite resin; although allowing for reproducible testing conditions, this does not reflect the wide variety of clinical situations where composite resins will be used clinically. Spectrophotometers were used to assess colour stability of the samples exposed to smoke which allow for accurate measurements of dental shades that may not be noticeable to the human eye^7^. The authors identified that only ten of the included studies discussed the clinical acceptability and perceptibility of colour change from their measurements. It would be useful to consider these findings against patient reported outcomes of RBC discolouration following smoking.

The majority of studies found CS results in higher levels of discolouration of RBCs compared to ENDS or THS. However, one study reported similar discolouration induced by CS and ENDS^8^. The smoke exposure protocol differed greatly between studies. Due to variation amongst individual users, it would be challenging to truly replicate the smoking exposure pattern although several of the studies also accounted for additional risks for RBC discolouration including dietary factors. Similarly, several of the studies accounted for the effect of saliva and toothbrushing; however, further evidence would be required as to how these factors would affect discolouration on a long-term basis.

This systematic review found that CS produces permanent RBC discolouration. The authors concluded THS and ENDS cause less discolouration than CS although evidence was limited and, in some areas, conflicting. Further research into the effects of CS, THS and ENDS on RBC colour stability is required.

Practice points

- Clinicians should inform patients that smoking conventional cigarettes may risk permanent discolouration of resin-based composites (RBCs).
- THS and ENDS seem to produce less discolouration of RBCs, which can be reversed, although evidence is limited.
- Explaining the potential risk of RBC discolouration may provide an opportunity to discuss smoking cessation with patients.
